# Amorphous Ag_2_S Micro-rods-Enhanced Fluorescence on Liquid Crystals: Cation-π Interaction-Triggered Aggregation-Induced Emission Effect

**DOI:** 10.1016/j.isci.2019.04.017

**Published:** 2019-04-15

**Authors:** Jianxin Kang, Jian Yu, Anran Li, Dongyu Zhao, Bin Liu, Lin Guo, Benzhong Tang

**Affiliations:** 1 Key Laboratory of Bio-Inspired Smart Interfacial Science and Technology, Ministry of Education, School of Chemistry, Beihang University, Beijing 100191, P.R. China; 2 Department of Chemistry, Hong Kong University of Science and Technology, Clear Water Bay, Kowloon, Hong Kong

**Keywords:** Microstructure, Nanoparticles, Optoelectronics

## Abstract

Aggregation-induced emission (AIE) system has long been regarded as a promising substitute to overcome the aggregation-caused quenching in traditional luminescent liquid crystals, which could further enhance its efficiency and application. However, due to the intrinsic weak interaction between hybrid components, heterogeneous inorganic materials-induced AIE process was rarely reported. In this study, trace amounts of amorphous Ag_2_S microrods and an AIE-active liquid crystalline compound tetraphenylethylene-propylbenzene (TPE-PPE) were proposed to construct additional intense interaction to trigger AIE effect. The enhanced concentration of unsaturated Ag ions and excess positive charge on Ag_2_S surface promote a cation-π interaction with TPE-PPE, leading to a 36-fold increase in fluorescence, which is predominately high in luminescent liquid crystal system. To the best of our knowledge, this is the first report of the AIE process activated by cation-π interaction. This novel approach would provide guidance to fabricate high-luminescence meso phases for future luminescent display device.

## Highlights

• Amorphous Ag_2_S micro-rods construct additional intense interaction to trigger AIE effect
• Unsaturated Ag ions and excess positive charge promote a cation-π interaction
• This heterogeneous inorganic material induced a 36-fold enhancement in fluorescence

## Graphical Abstract

**Figure:**
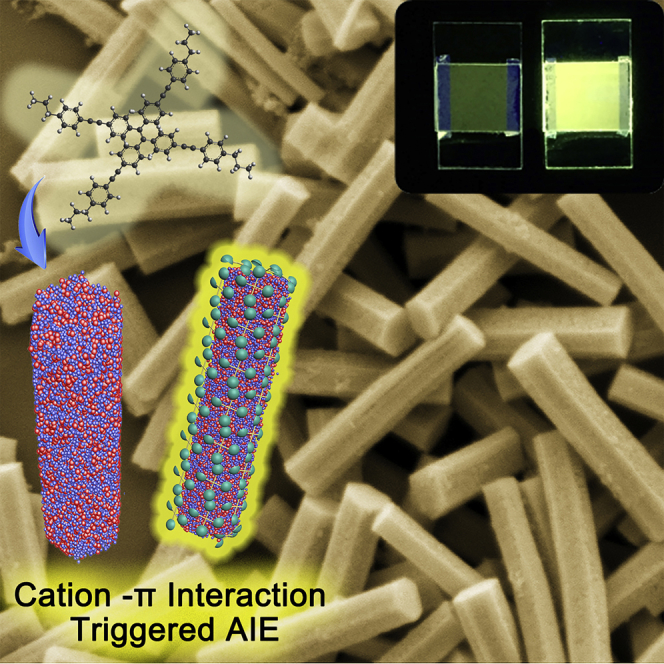

## Introduction

Combining intrinsic light emission and unique self-organization properties, luminescent liquid crystals (LE-LCs) have currently attracted extensive research attentions, not only for overcoming the disadvantages of conventional passive display techniques, such as low brightness and low energy efficiency, but also due to the capability of emitting linear or circular polarized light, which is of paramount importance in applications of versatile potential optoelectronic devices (Grell and Bradley, 1999, Liu et al., 2009, O'Neill and Kelly, 2003, Vijayaraghavan et al., 2008, Yuan et al., 2012). However, the luminescent performance in liquid crystalline phase has been confined by the intense intermolecular interactions among the extremely ordered LC molecules for formation of π-π stacking interactions, which would lead to intrinsic self-quenching of a majority of luminogens, described as aggregation-caused quenching (ACQ) effects (Chiang et al., 2008, Grimsdale et al., 2009, Jiang et al., 2017). Recently, a novel photophysical phenomenon of aggregation-induced emission (AIE) was discovered based on some propeller-like organic molecules (Huang et al., 2018, Li et al., 2018, Luo et al., 2001, Sugiuchi et al., 2017, Zhao et al., 2018). Instead of the normally observed quenching in “conventional” luminophores, the restriction of intramolecular motion (RIM) (Chi et al., 2012, Mao et al., 2019, Mei et al., 2015, Zhang et al., 2015b) in aggregation state would intensify their fluorescence, transforming the weakly luminescent chromo-gens into fierce luminophores. The non-traditional systems, AIE-gens, thus provided a possibility to solve the conflicts between fluorescence quenching caused by the aggregation and the requirement of self-organization for LCs, becoming promising candidates for the evolution of novel LE-LCs (Bui et al., 2016, Feng et al., 2017, Jiang et al., 2018, Kim et al., 2014, Lu et al., 2016, Park et al., 2014, Pathak et al., 2016, Tanabe et al., 2012, Yu et al., 2013).

Even though the factors influencing AIE process have been fully explored, establishing new mechanisms to trigger AIE action or a novel AIE system are still desired. For example, relative to pure organic AIE systems, research in inorganic-organic hybrid systems was still insufficient (Hu et al., 2011, Li et al., 2013, Mahtab et al., 2011, Wei et al., 2014, Zhang et al., 2014a, Zhang et al., 2014b). As was reported, metal nanoparticles have special application in fluorescence enhancement owing to electric field effect and the induced plasmon effect (Camposeo et al., 2015, Pompa et al., 2006). With these two approaches, the emission intensities of fluorophores often show a significant increase proximal to the metallic nanostructures. However, inorganic nanoparticle-induced fluorescence enhancement governed by a more general mechanism has been rarely observed thus far. In inorganic nanomaterials, surface defects always provide optimal environment for reactant adsorption, which was induced by the lower coordination number than that of bulk phase (Ni and Wang, 2015, Xie et al., 2013a, Xie et al., 2013b). Amorphous materials that contain intrinsically disordered atomic arrangement and lower coordination number could be a potential verifiable research platform for unsaturated environment (Ye et al., 2017, Zhao et al., 2017).

In this work, we explore a novel system of invoking amorphous Ag_2_S micro-rods and an AIE-active liquid crystalline compound tetraphenylethylene-propylbenzene (TPE-PPE) into the nematic LC medium to promote the enhancement of emission in LCs, by taking the advantage of cation-π interactions between the amorphous Ag_2_S and the AIE-LC molecules. The S-defect-rich feature of amorphous Ag_2_S micro-rod leads to enhanced surface concentration of unsaturated Ag ions and excess surface positive charge, which respectively provided adsorption sites and interaction for the adsorption of TPE-PPE. This intensive electrostatic interaction between excess cation-modified inorganic nanomaterials and conjugated electron-rich organic system restricted the intramolecular motions of TPE-PPE in the liquid crystal medium, with the synergistic combination of concentration effect leading to an ∼36-fold fluorescence enhancement in LCs. Even though cation-π interaction has been universally found to be essential in molecular recognition, organic synthesis, host-guest complexation, supramolecular chemistry, and biology (Craven et al., 2016, Dougherty, 2013, Mahadevi and Sastry, 2013), to the best of our knowledge, no article concerning cation-π interaction-activated AIE process has been reported.

## Results

**Figure 1:**
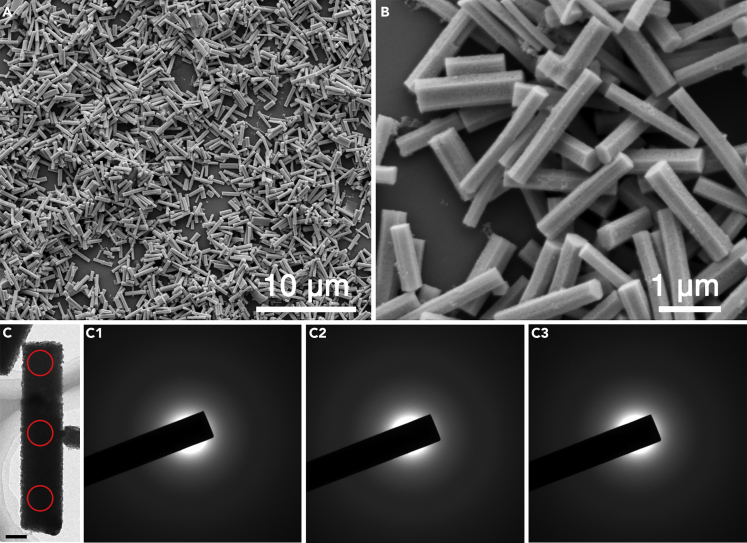
Morphology and Structure of Amorphous Ag_2_S (A and B) Scanning electron microscopic images of amorphous Ag_2_S micro-rods under different magnification. (C) Transmission electron microscopic images and corresponding selected area electron diffraction patterns (C_1_–C_3_) of the circled regions in (C). Scale bar, 100 nm in (C).

As was discussed, RIM in aggregated state is key for AIE (Hu et al., 2014, Zhang et al., 2014c, Zhang et al., 2014d).To provide a model with plentiful unsaturated adsorption sites, an amorphous material is synthesized through a two-step solvent precipitation and decomposition method (See Supplemental Information). As shown in Figures 1A and 1B, under different magnification of scanning electron microscope, a remarkably uniform micro-rod with clear hexagonal prism morphology was synthesized. The average diameter was ∼0.3 μm, and the length was ∼2 μm. On the contrary, in transmission electron microscopy, the typical selected area electron diffraction patterns (Figures 1C_1_–1C_3_) circled in different parts of a prism show dispersive haloes, which are typical amorphous characters. X-ray diffraction pattern exhibited only a broad hump instead of sharp peaks (Figure S1), further confirming the amorphous structure.

The chemical component of this micro-rod was confirmed to be Ag_2_S by X-ray photoelectron spectroscopy (XPS) in Figure S2. However, the surface atomic ratio of Ag and S exhibited by XPS was up to 6:1, much higher than the stoichiometric proportion of Ag_2_S; it revealed that Ag atoms were partly enriched on the surface. To obtain more precise information, Auger electron spectroscopy (AES) was also employed, which even displayed a higher surface sensitivity than XPS (Figure S3). For Ag M4N45N45 Auger-electron line, a tiny shoulder peak at 358.3 eV emerged beside the typical Ag (I) peak at 355.8 eV (Gaarenstroom and Winograd, 1977). At the same time, the Ag M4N45N45 3P Auger-electron line was also broadened from the standard Ag (I) peak at 350.3 eV–∼352.0 eV. Both of them appeared at higher kinetic energy, indicating a lower oxidation state of Ag. Combined with the higher atomic ratio of Ag and S, S atom vacancies were predicted. To judge its unsaturated environment, the S-defect was evaluated using electron paramagnetic resonance (EPR). EPR spectra provided sufficient evidences for probing sulfur vacancies with obvious signals at 320–324 mT, which were identified as electrons trapped on sulfur vacancies (Figure S4). Sulfur vacancy-induced excess surface positive charge was also detected by zeta potential, whose corresponding result showed a large positive as high as 45 ± 4 mV (Figure S5), indicating plentiful positive charges. The enhanced surface concentration of unsaturated Ag ions and excess positive charge on the surface made it a potential platform for molecular connection and electric charge transformation.

**Figure 2:**
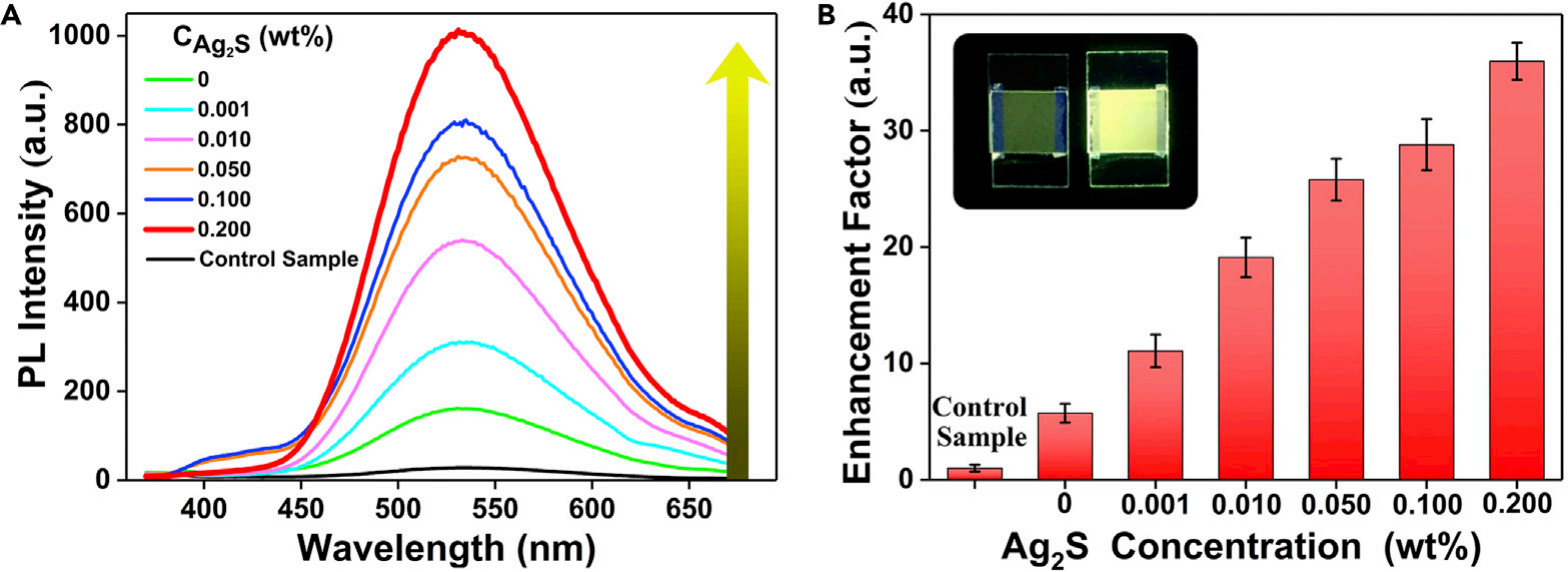
Amorphous Ag_2_S-Enhanced Fluorescence in Luminescent Liquid Crystals (A and B) (A) PL spectra and (B) fluorescence enhancement factor histograms of the SLC/TPE-PPE (100/0.20 wt %) with different Ag_2_S concentration doping. Excitation wavelength: 340 nm. PL spectra of the control sample (100/0.01 wt %/wt %) are also shown for comparison. The inset in (B) shows photographs of control sample and the optimal sample with largest fluorescence intensity in LC cells.

Then, the luminescent LC systems composed of the LC host, an AIE-active luminogen, and amorphous Ag_2_S were fabricated and evaluated through photoluminescence (PL) spectroscopy. Here, the positive LC is nematic LC, SLC1717, and the AIE-LC was TPE-PPE (Zhao et al., 2015, Zhao et al., 2016), whose molecular structure is shown in Figure S6. As shown in Figure S7, the homogeneous micrographs of polarization microscope exhibited that the alignment of LCs was not disturbed in the presence of Ag_2_S at the concentration of 0.2 wt %. The intrinsic AIE effect was first studied, in which 0.20 wt % TPE-PPE led to approximately 5.70-fold higher optimal emission intensity than 0.01 wt % compositions (Figures S8A and S8A′). Fixing the optimal concentration of TPE-PPE at 0.20 wt %, PL measurements on varying concentrations of amorphous Ag_2_S displayed a maximum PL intensity at an Ag_2_S concentration of 0.20 wt %, with 6-fold enhancement (Figure 2A). It was an exciting 36-fold enhancement than the primal control sample with 0.01 wt % TPE-PPE compositions as shown in Figure 2B. Apart from the PL spectroscopy, the quantum yield of the system also exhibited the same tendency as PL intensity, which was enhanced along with the increase of Ag_2_S doping (Table S1). This aggregation-induced enhancement was attributed to the intensive interaction of amorphous Ag_2_S and AIE-gens, because the large enhancement could also be repeated in glycerinum-tetrahydrofuran (v/v 1:1) solvent (Figure S9). Moreover, the fluorescence stability of the composites was also evaluated by monitoring the changes in PL intensity. Under ambient conditions, the LC cells were found to be quite stable for at least 1 month and almost no variations were observed in PL performance. This result was fascinating when compared with ACQ effects in fluorescein (Figures S8B and S8B′), in which the rigid planar aromatic structure (Figure S10) would lead to π-π stacking interaction in LC phase aggregation. As was compared, if Ag_2_S was added into the SLC1717/fluorescein composite (Figure S11), its emission is significantly quenched.

**Figure 3:**
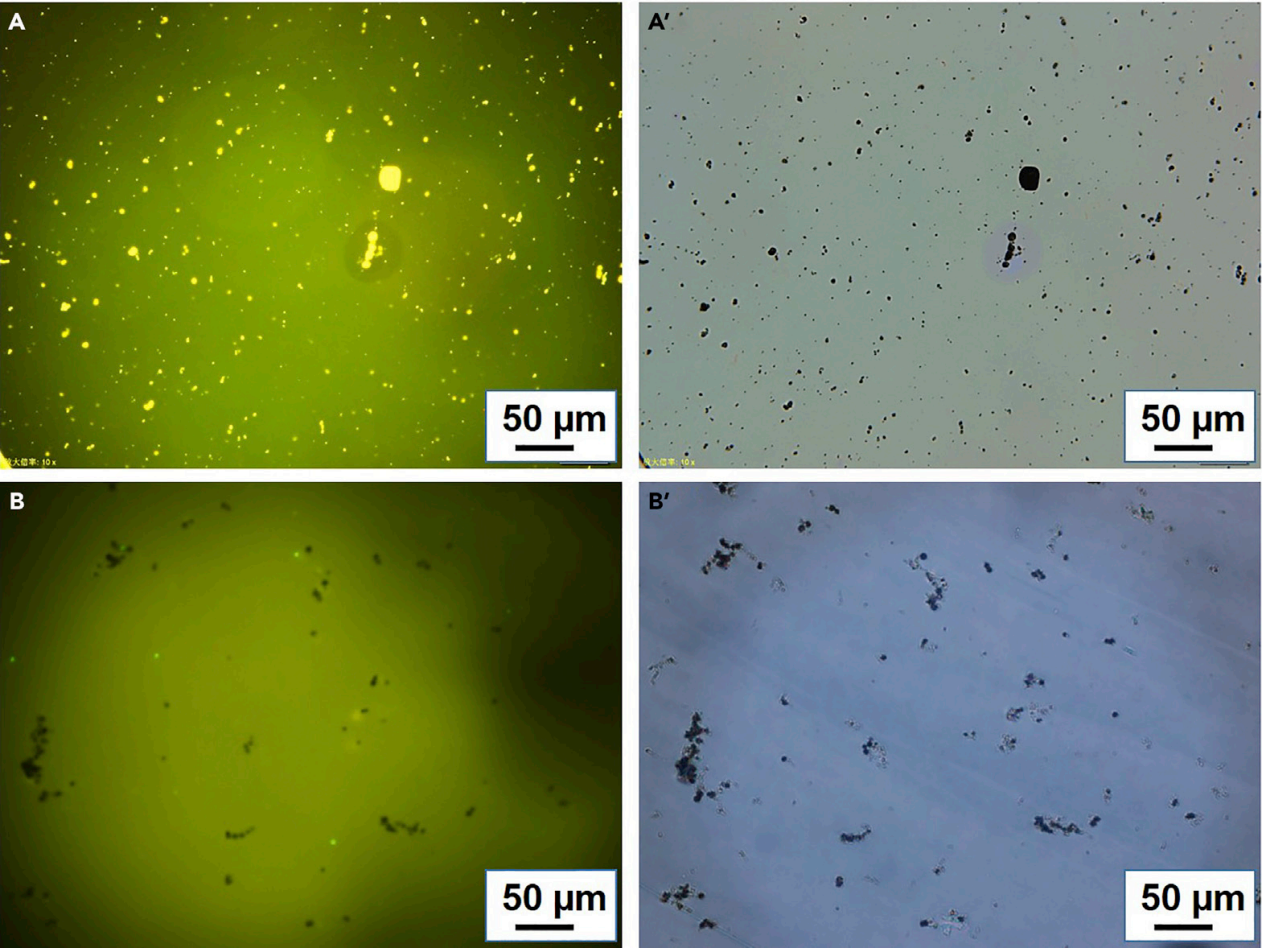
Comparison of Ag_2_S Induced AIE and ACQ under Fluorescence Microscope (A and A′) Photographs of the LC test cell filled with LC-Ag_2_S-TPE-PPE. (B and B′): Photographs of the LC test cell filled with LC-Ag_2_S-fluorescein. (A and B) Images were taken under fluorescence microscope.

Till now, studies on inorganic-organic hybrid systems regarding fluorescence enhancement are insufficient (Hu et al., 2011, Xu et al., 2011). To reveal the mechanism of Ag_2_S-induced fluorescence enhancement, the fluorescent performances of LCs-TPE-PPE-Ag_2_S and the LCs-fluorescein-Ag_2_S systems were compared using fluorescence microscope. As shown in Figures 3A and 3A′, the LC cell composed of LCs-Ag_2_S-TPE-PPE was observed to be light emitting and the Ag_2_S micro-rods doped in it exhibited higher brightness than the luminescent LC host. In contrast, the Ag_2_S micro-rods doped in the LCs-Ag_2_S-fluorescein system were dimmer than the light-emitting LCs as, shown in Figures 3B and 3B′. Therefore the fluorescence enhancement should be attributed to the adsorption of AIE-active TPE-PPE to the doped Ag_2_S. Once dispersed into the LC medium, Ag_2_S micro-rods would absorb fluorescent dyes and lead to the second aggregation of fluorescent dye in local environment, which further enhanced the emission of luminescent LC mixtures.

For amorphous inorganic nanomaterials, surface defects always provide optimal environment for reactant adsorption (Zhang et al., 2015a). In our system, the sulfur vacancies of amorphous Ag_2_S would induce unsaturated coordinated structure, which would be an appropriate environment for TPE-PPE abortion, while positive charges coming from the surplus Ag would also afford the electron environment (Craven et al., 2016) for the link of π-electron circulation of TPE-PPE. To confirm the interaction, the amorphous Ag_2_S micro-rod was compared with crystal Ag and AgNO_3_ nanoparticles under the same modified condition by AES spectra (Figure S12). It was clear that Auger-electron line of Ag(I) parts of TPE-PPE-modified amorphous Ag_2_S micro-rods exhibited a tiny shift to the high kinetic energy. It means the conjugate electrons of TPE-PPE indeed partly transferred to the surficial Ag(I) ions, leading to a lower oxidation state and a lower binding energy of Ag(I). In contrast, crystal Ag and AgNO_3_ showed almost the same spectra with their modified samples, and it was also embodied in their PL spectra, which displayed a maximum enhancement of PL intensity of about 1.7 times (Figure S13).

**Figure 4:**
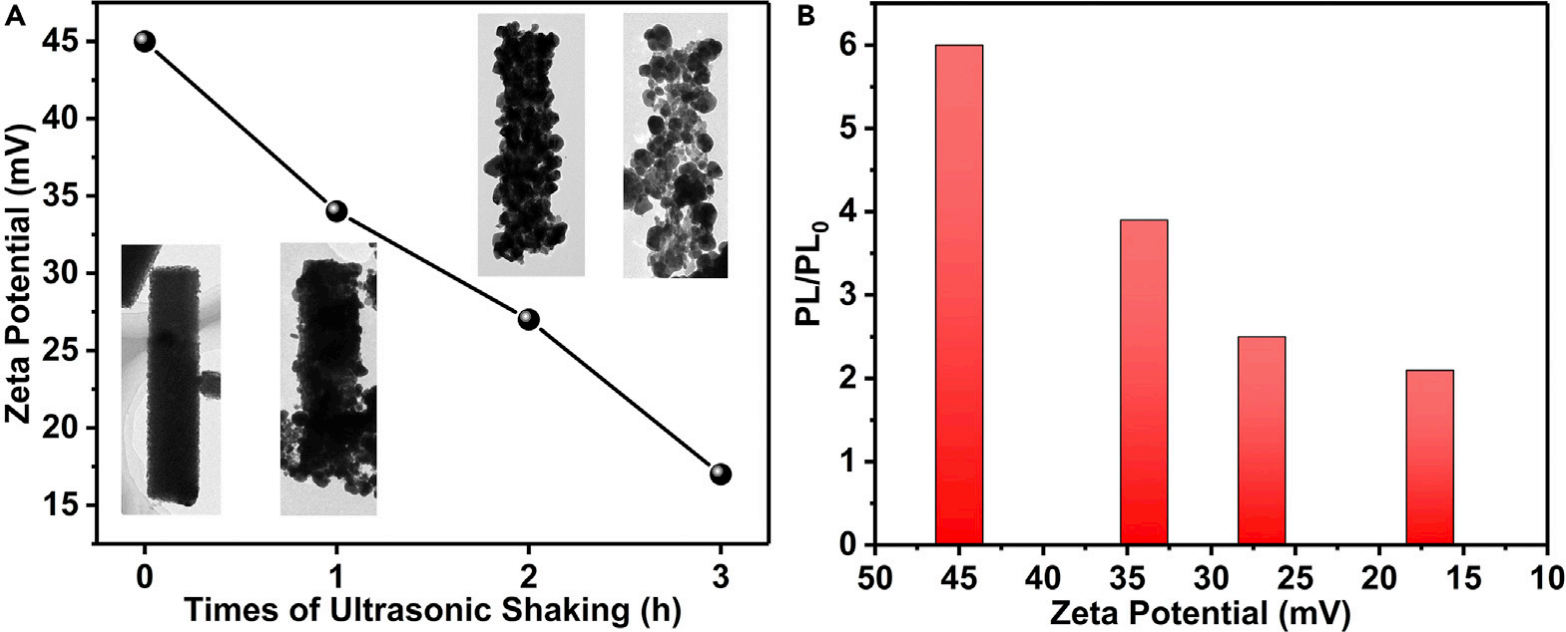
The Corresponding Relation of Ag_2_S Crystallinity, Zeta Potential, and Fluorescence Intensity (A) The zeta potential and corresponding morphology evolution along with crystalline process induced by ultrasonic shaking. (B) Relative fluorescence intensity of LC cells filled with emissive LC composites against the zeta potential of Ag_2_S. Emissive LC composite = LC1717 + 0.2 wt % TPE-PPE + 0.2 wt % processed Ag_2_S. Here, the LC cell filled with 0.2 wt % TPE-LC1717 was used as a standard (PL_0_) sample.

Thus the amorphous Ag_2_S micro-rods could be an excellent second-level assembly core to absorb TPE-PPE. To confirm that the amorphous structure induced surface positive electric charge, a series of materials with different crystallinities was prepared through long-time ultrasonic shaking. After 3-h treatment, the amorphous micro-rods would turn to hollow crystal Ag_2_S rods, which were assembled by abundant 50- to 100-nm nanospheres (Figure S14). Products of different stage were measured by zeta potential to quantificationally evaluate the electric charge on the surface. It is clear that along with the crystallization process, zeta potential persistently reduces from ∼45 mV to ∼15 mV, illustrating the fading away of surface positive electric charges (Figure 4A). Then the corresponding fluorescence intensity (PL/PL_0_) of the LCs-TPE-PPE-Ag_2_S composites with different degrees of crystallinity of Ag_2_S was monitored (Figure 4B). The relative fluorescence intensity exhibits a gradual increase with the increase of the zeta potential of the Ag_2_S, revealing the essential role of surface electric charges of doped Ag_2_S on the fluorescence activities of TPE-PPE. To verify the interaction between surface positive charge and conjugated electron-rich aromatic system, a rough model was established to evaluate the single point energies of Ag atom, Ag (I) ion, Ag_2_S cluster, and phenyl using the first principles calculation method (Table S1). When coupled with the phenyl ring, the binding energies were calculated to increase along with the increase of the electric charge (Figure S15). Evidently, this calculation result suggests that the existence of cation-π interactions played the dominant role between inorganic materials and conjugated electron-rich TPE-PPE.

## Discussion

**Scheme 1:**
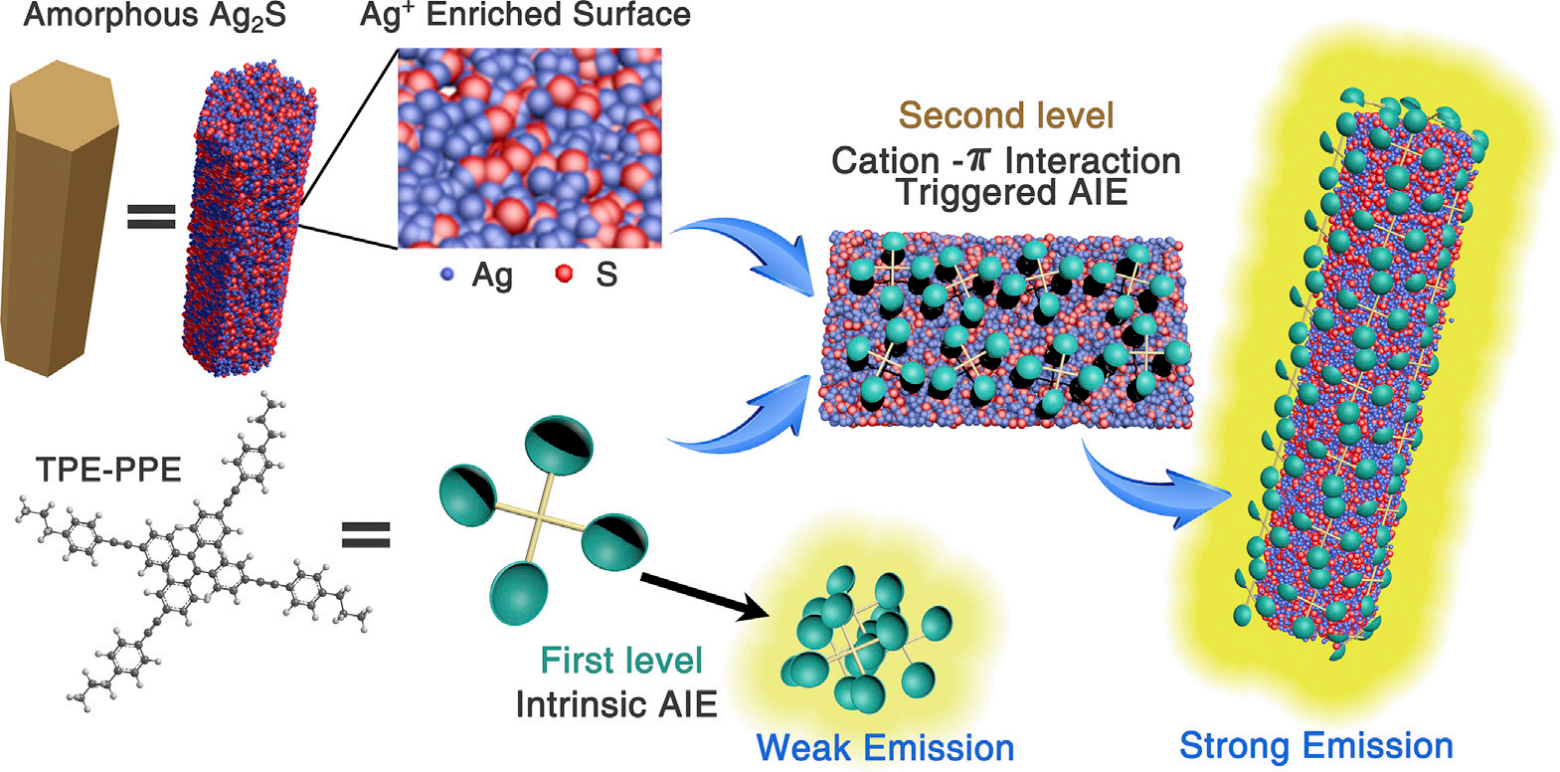
Schematic Illustration of Proposed Mechanism for Amorphous Ag_2_S-Induced AIE Behavior through Ag-π Interaction between Ag_2_S Surface and TPE-PPE

Based on our experimental and calculation results, a proposed mechanism for amorphous Ag_2_S micro-rods-induced emission enhancement of AIE-LC system has been extracted (Scheme 1). The employed amorphous Ag_2_S will absorb the fluorescent dyes (TPE-PPE) in the LC medium, leading to the aggregation of the AIE-gen; it was attributed to the enhanced surface concentration of unsaturated Ag ions and excess positive charge on the surface of the amorphous Ag_2_S micro-rods, which, respectively, provided optimized adsorption sites and adsorption interaction, and promoted surface Ag ions to form cation-π interaction with fluorescence dyes containing conjugated π-electrons. As a result, the RIM process was triggered and strong emission was turned on. On the contrary, when the amorphous Ag_2_S was doped into LC-fluorescein system, the fluorescence was quenched, owing to the similar cation-π interaction between Ag_2_S and aromatic rings of fluorescence.

In summary, novel amorphous Ag_2_S micro-rods were synthesized to be a potential aggregation center to explore if inorganic nanoparticles improved the luminescence properties of AIE-active LCs. With trace amounts of amorphous Ag_2_S and AIE dye TPE-PPE introduced into nematic LCs, an ∼36-fold PL intensity enhancement was obtained. To the best of our knowledge, the enhancement factor is predominately high among those reported for luminescent LCs. The intensive electrostatic interaction between excess cation of amorphous inorganic nanomaterials and conjugated electron-rich organic system played a dominant role in the emission enhancement of TPE-PPE in the nematic LC host. Extraordinarily, it is the first report to applying cation-π interaction into the fluorescence enhancement of LC systems. This report will contribute to open up a new prospect of light-emitting LCs by introducing micro-materials into LC systems, leading to more potential applications for fabricating LC dispalys.

### Limitations of the Study

We showed the intense interaction between amorphous Ag_2_S micro-rods and the AIE dye TPE-PPE through the obvious luminescence enhancement under fluorescence microscope. However, it is very difficult to simulate this process. Even though the atomic-level model built in this work is able to give a qualitative description to evaluate the cation-π interaction, a more perfect model is needed in the future for a better understanding.

## Methods

All methods can be found in the accompanying Transparent Methods supplemental file.

## Supplemental Information

Document S1. Transparent Methods, Figures S1–S15, and Tables S1 and S2
