# Supplementary material for: Amorphous Ag2S Micro-rods-Enhanced Fluorescence on Liquid Crystals: Cation-π Interaction-Triggered Aggregation-Induced Emission Effect
Source: iScience. 2019 Apr 15;15:119–26. doi: 10.1016/j.isci.2019.04.017 (PMC6495463; doi:10.1016/j.isci.2019.04.017)
Supplement: Document S1. Transparent Methods, Figures S1–S15, and Tables S1 and S2 [file mmc1.pdf]

ISCI, Volume *15*

Supplemental Information

Amorphous Ag_2S Micro-rods-Enhanced Fluorescence on Liquid Crystals: Cation-π Interaction-Triggered Aggregation-Induced Emission Effect

Jianxin Kang, Jian Yu, Anran Li, Dongyu Zhao, Bin Liu, Lin Guo, and Benzhong Tang

Supporting Information

Amorphous Ag_2S Microrods Enhanced Fluorescence on Luminescent Liquid Crystals: Cation-π Interaction Triggered AIE Effect

Jianxin Kang, [1,4] Jian Yu, [1,4] Anran Li, [1] Dongyu Zhao, [1,]* Bin Liu[1], Lin Guo, [1,3,]* and Benzhong Tang[2,]*

[1] Beijing Advanced Innovation Center for Biomedical Engineering，School of Chemistry, Beihang University, Beijing 100191, P.R. China

[2] Department of Chemistry, Hong Kong University of Science and Technology, Clear Water Bay, Kowloon, Hong Kong

[3] Lead Contact

[4] These authors contributed equally to this work.

*Correspondence: guolin@buaa.edu.cn

This PDF files includes:

Fig. S1. XRD pattern of amorphous Ag_2S micro-rods.

Fig. S2. XPS spectra of amorphous Ag_2S micro-rods.

Fig. S3. AES spectra of Ag $M_4N_{45}N_{45}$ Auger-electron line for amorphous Ag_2S micro-rods.

Fig. S4. EPR spectra of amorphous Ag_2S micro-rods.

Fig. S5. Zeta potential distribution of amorphous Ag_2S micro-rods dispersed in deionized water.

Fig. S6. Chemical structure of TPE-PPE.

Fig. S7. POM micrographs of the LC/TPE-PPE/Ag_2S (0.20 wt%) composite.

Fig. S8. PL spectra of the LC cells composed of nematic SLC1717 and TPE-PPE (fluorescein).

Table S1. Result of the quantum yield of the composites (SLC1717+0.2wt% TPE-PPE+Ag_2S).

Fig. S9. PL spectra of TPE-PPE with Ag_2S in glycerinum-THF solvent (v/v 1:1).

Fig. S10. Chemical structure of fluorescein.

Fig. S11. PL spectra of the sample SLC1717/fluorescein (100/0.20 wt%/wt%) composite doping Ag_2S with concentration of 0 and 0.20 wt%.

Fig. S12. AES spectra of amorphous Ag_2S micro-rods, crystal Ag, crystal $AgNO_3$ nanoparticles and their modified samples.

Fig. S13. PL spectra of crystal Ag and $AgNO_3$ nanoparticles doped into the SLC1717/TPE-PPE composite.

Fig. S14. Morphology and structure of crystallized amorphous Ag_2S.

Table. S2. Single point energies of the elements in the calculation by first principle calculation method.

Fig. S15. Structures and binging energy of Ag atom, Ag(I) ion and Ag_2S cluster with the aromatic ring.

Transparent Methods

Supplemental Figures

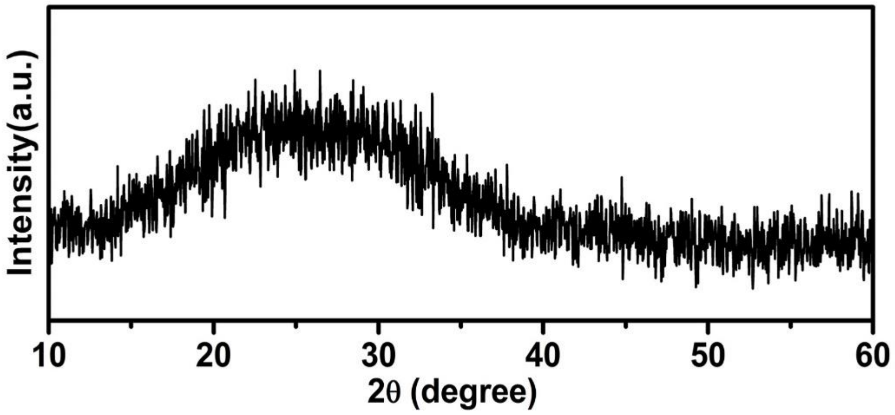

Figure S1. XRD pattern of amorphous Ag_2S micro-rods, related to Figure 1. It exhibited only a broad hump instead of sharp peaks, further confirmed the amorphous structure.

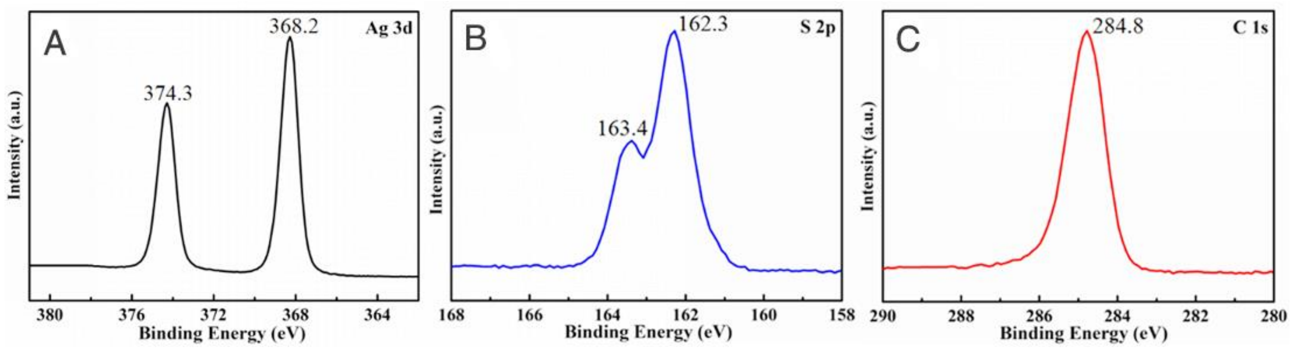

Figure S2. XPS spectra of (A) Ag 3d, (B) S 2p and (C) C 1s for amorphous Ag_2S micro-rods, related to Figure 1. The Ag $3d_{5/2}$ and Ag $3d_{3/2}$ binding energies were located at 368.2 eV and 374.3 eV respectively, which indicates the Ag (I) species. While, the S $2p_{3/2}$ and S $2p_{1/2}$ peaks appeared at 162.3 eV and 163.5 eV, respectively, a typical inorganic covalent bond environment for S. C 1s spectrum was used for calibration. Thus, the as-prepared micro-rods should be amorphous Ag_2S.

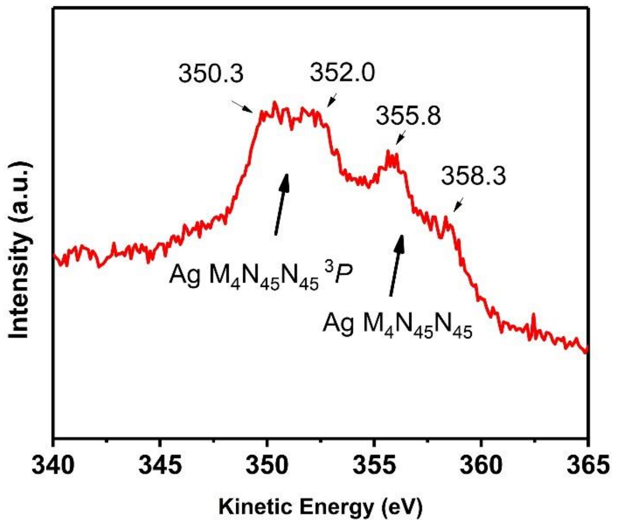

Figure S3. AES spectra of Ag $M_4N_{45}N_{45}$ Auger-electron line for amorphous Ag_2S micro-rods, related to Figure 1. A tiny shoulder peak at 358.3 eV was emerged beside the typical Ag (I) peak at 355.8 eV. At the same time， the Ag $M_4N_{45}N_{45}$ ^3P Auger-electron line was also broadened from the standard Ag (I) peak at 350.3 eV to ~352.0 eV. Both appeared at higher kinetic energy, indicated a lower oxidation state of Ag.

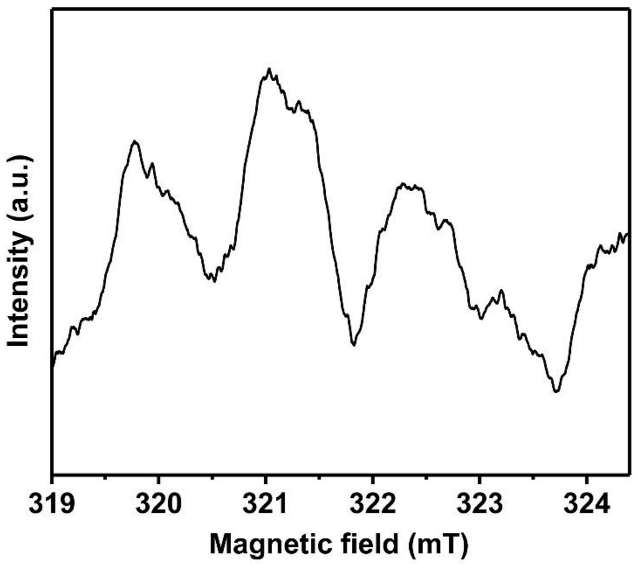

Figure S4. Electron paramagnetic resonance (EPR) spectra of amorphous Ag_2S micro-rods, related to Figure 1. EPR spectra provided sufficient evidences for probing sulfur vacancies with obvious signals at about 319~324 mT, which were identified as electrons trapped on sulfur vacancies.

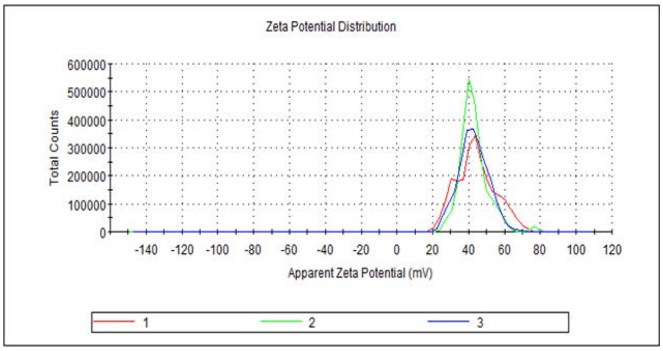

Figure S5. Zeta potential distribution of amorphous Ag_2S micro-rods dispersed in deionized water, whose corresponded result showed a large positive as high as 45±4 mV (three independent experiments showed in three different colors), indicating a plentiful of positive charge, related to Figure 1.

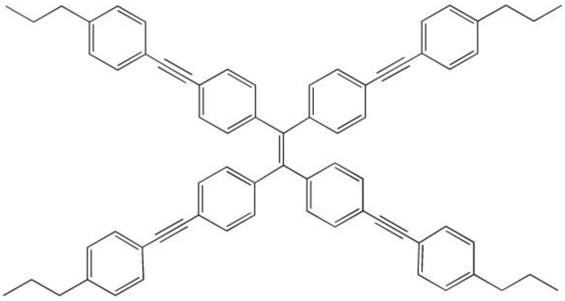

Figure S6. The chemical structure of TPE-PPE, related to Figure 2. In this molecule, it has a conjugation structure of four mesogenic units and a TPE core, exhibiting both AIE behaviors and liquid crystalline properties. Four mesogenic units are linked to a central olefin stator through single-bond axes, respectively.

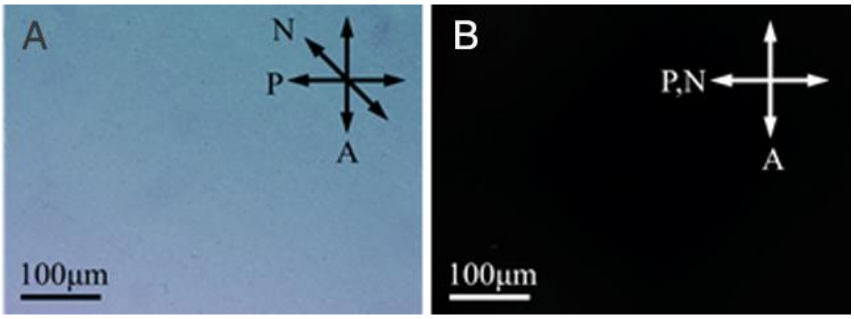

Figure S7. POM micrographs of the LC/TPE-PPE/Ag_2S (0.20 wt%) composite with N at (a) 45° and (b) 0° to P, related to Figure 2. (A: analyzer, P: polarizer, N: director of LCs).

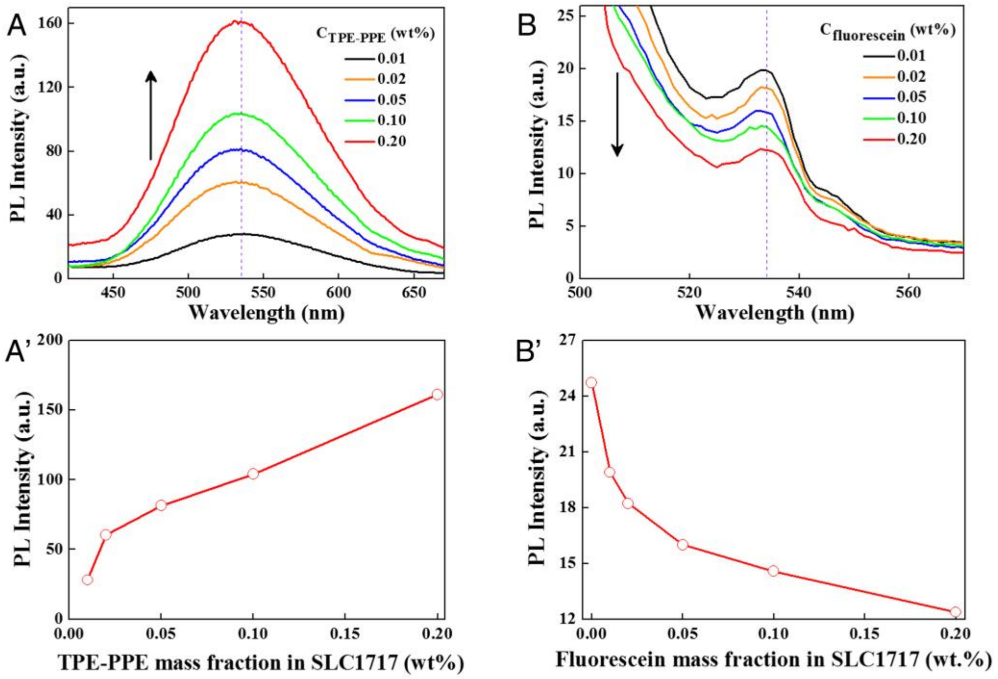

Figure S8, related to Figure 2 and 3**.** (A) Normal PL spectra of the LC cells composed of nematic SLC1717 and TPE-PPE with different concentrations. Excitation wavelength: 340 nm. (A') PL intensity values at ~535 nm versus the concentrations of TPE-PPE in SLC1717. (B) PL spectra of the LC cells composed of SLC1717 and fluorescein with the identical concentrations. Excitation wavelength: 489 nm. (B') PL intensity values at ~535 nm versus the concentrations of fluorescein in in SLC1717. It is worthy to notice that LCs containing various concentrations of TPE-PPE exhibited identical emission profile and wavelength (λ_{max} = 535 nm), excited by 340 nm ultraviolet light.

Distinctly, it can be observed that all the luminescent liquid crystal samples displayed a fairly broad PL spectrum in the visible regions from 450 nm to 650 nm. Moreover, the fluorescence behavior varied directly with concentration of TPE-PPE in LC phases. The emission of the LC host dissolved with 0.01 wt% TPE-PPE was so faint that its PL spectra appeared a dinky peak. However, the emission of the "guest-host" devices was switched on along with the concentration of doped TPE-PPE increased. With TPE-PPE fraction further increased, the light emission steadily intensified and PL peak appeared with no shift. As the concentration of luminescent TPE-PPE increased to 0.20 wt%, the optimal emission intensity at 535 nm was approximately 5.70-fold higher than that in 0.01 wt% TPE-PPE compositions. It was due to the different aggregate level derived from different concentrations of TPE-PPE in mesophases. In TPE-PPE, four mesogenic units are linked to a central olefin stator through single-bond axes, respectively. At low concentration of TPE-PPE in LC matrix, TPE-PPE molecules were almost isolated and would suffer from vibrant intramolecular rotations, leading to radiation-less relaxation of the excitons and emitting very weak light. However, along with the concentration of TPE-PPE in the viscous LC host was increased and the aggregation were formed,

the accompanying RIM and highly distorted molecular structures would hinder the intermolecular π-π stacking interaction of LCs. Therefore, higher concentrations of TPE-PPE were more conducive to overcome rotation barrier, which would bring a more efficient luminescence in LC states. While, the aggregation of fluorescein presented a rather noteworthy critical concentration quenching effect, resulting in the emission quenching of the luminophores in liquid crystal states. In a preliminary comparison, we utilized a prototypical fluorophore, fluorescein to study the AIE effects from the reverse side. Apparently, it could be noticed that the emission intensity of liquid crystal/fluorescein mixtures was intensively decreased when fluorescein, an ACQ dye, is progressively added into liquid crystal matrix. When the concentration of fluorescein rose to 0.20 wt%, the ACQ effects became visibly discriminable and the emission got exceedingly faint. In contrast to the RIR conformations of TPE-PPE, the rigid planar aromatic structures of fluorescein lead to π-π stacking interaction in liquid crystal phases. The excited states after absorbed energy would decay back to the ground states via non-radiative intermolecular energy transfer.

Table S1. Result of the quantum yield of the composites (SLC1717+0.2wt% TPE-PPE+Ag_2S), , related to Figure 2.

Ag_2S amount （wt%）	0	0.001	0.01	0.05	0.10	0.20
QY (quantum yield)	13.51%	14.00%	19.27%	27.09%	31.33%	38.22%

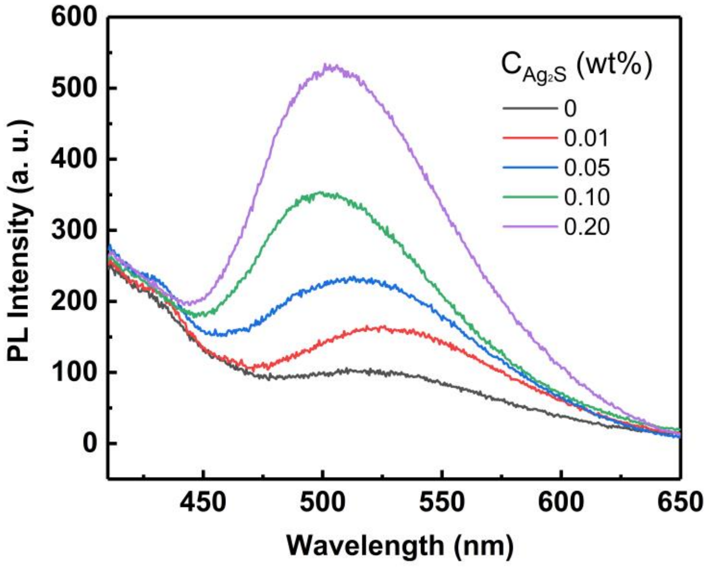

Figure S9. PL spectra of TPE-PPE (0.20 wt%) with different Ag_2S concentrations doping in glycerinum-THF solvent (v/v 1:1). Excitation wavelength: 340 nm, related to Figure 2.

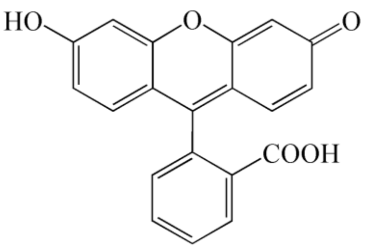

Figure S10. The chemical structure of fluorescein, which displayed a rigid planar aromatic structure, related to Figure 2 and 3.

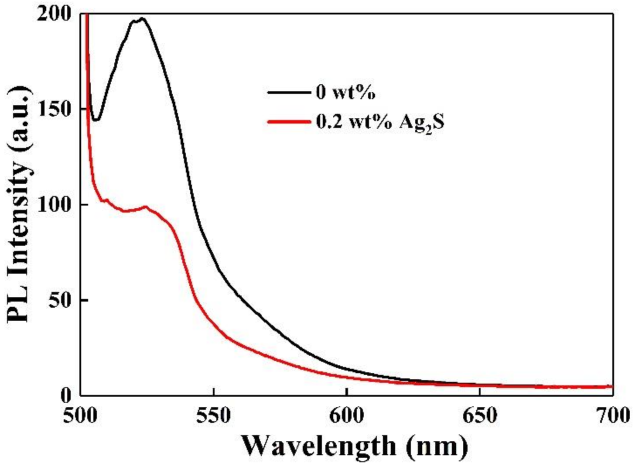

Figure S11. PL spectra of the sample SLC1717/fluorescein (100/0.20 wt%) composite doping Ag_2S with concentration of 0 and 0.20 wt%, related to Figure 2 and 3. Excitation wavelength: 489 nm.

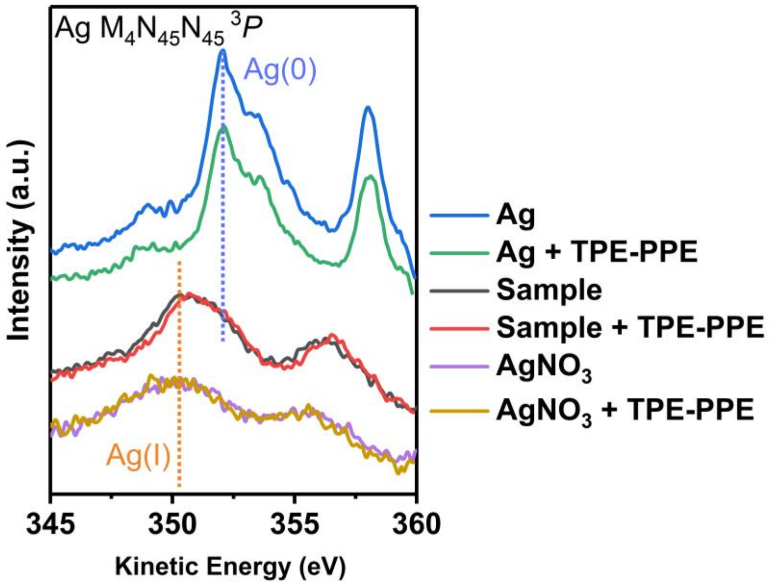

Figure S12. AES spectra of Ag $M_4N_{45}N_{45}$ Auger-electron line for amorphous Ag_2S micro-rods, crystal Ag, crystal $AgNO_3$ nanoparticles and their modified samples, related to Figure 1, 2 and 3. Compared with pure crystal Ag/$AgNO_3$ and TPE-PPE modified Ag/$AgNO_3$, Auger-electron line of Ag(I) parts of TPE-PPE modified amorphous Ag_2S micro-rods showed a tiny shift to the high kinetic energy. It means the conjugate electrons of TPE-PPE indeed partly transferred to the surficial Ag(I) irons, leading to a lower oxidation state and a lower binding energy of Ag(I).

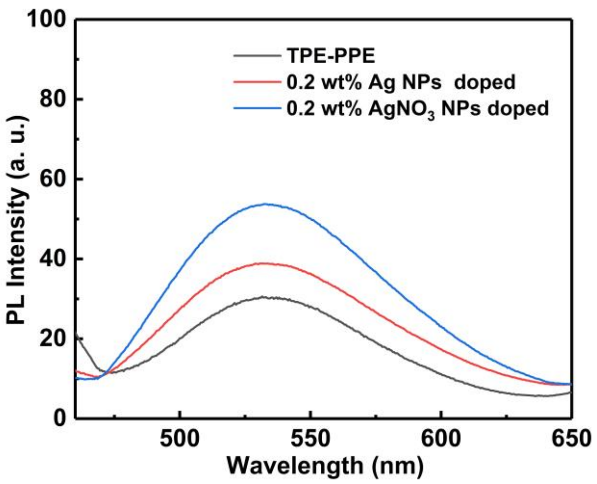

Figure S13. PL spectra of 0.20 wt% crystal Ag and $AgNO_3$ nanoparticles (NPs) doped into the SLC1717/TPE-PPE (100/0.20 wt%) composite, related to Figure 2 and 3. Compared with the original composite, the fluorescence enhancement of Ag and $AgNO_3$ NPs were much lower than amorphous Ag_2S micro-rods. Excitation wavelength: 340 nm.

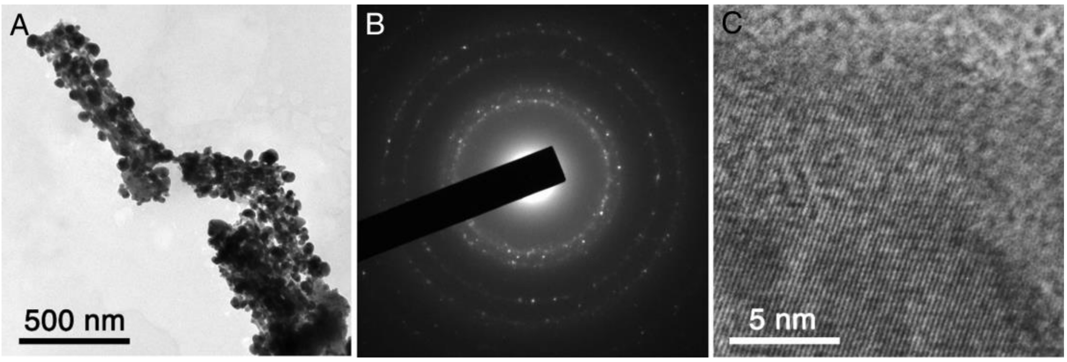

Figure S14. (A) TEM image, (B) SAED pattern and (C) HRTEM of the product under ultrasonic shaking for 3 h, related to Figure 4.

Table S2. Single point energies of the elements in the calculation by first principle calculation method, related to Figure 3 and 4.

	Single Point Energy (Kcal/mol)
Ag atom	-9.186*10^4
Ag (I) ion	-9.169*10^4
Ag_2S cluster	-4.330*10^5
Benzene ring	-1.453*10^5
Ag-benzene	-2.371*10^5
Ag (I)-benzene	-2.498*10^5
Ag_2S cluster-benzene	-4.333*10^5

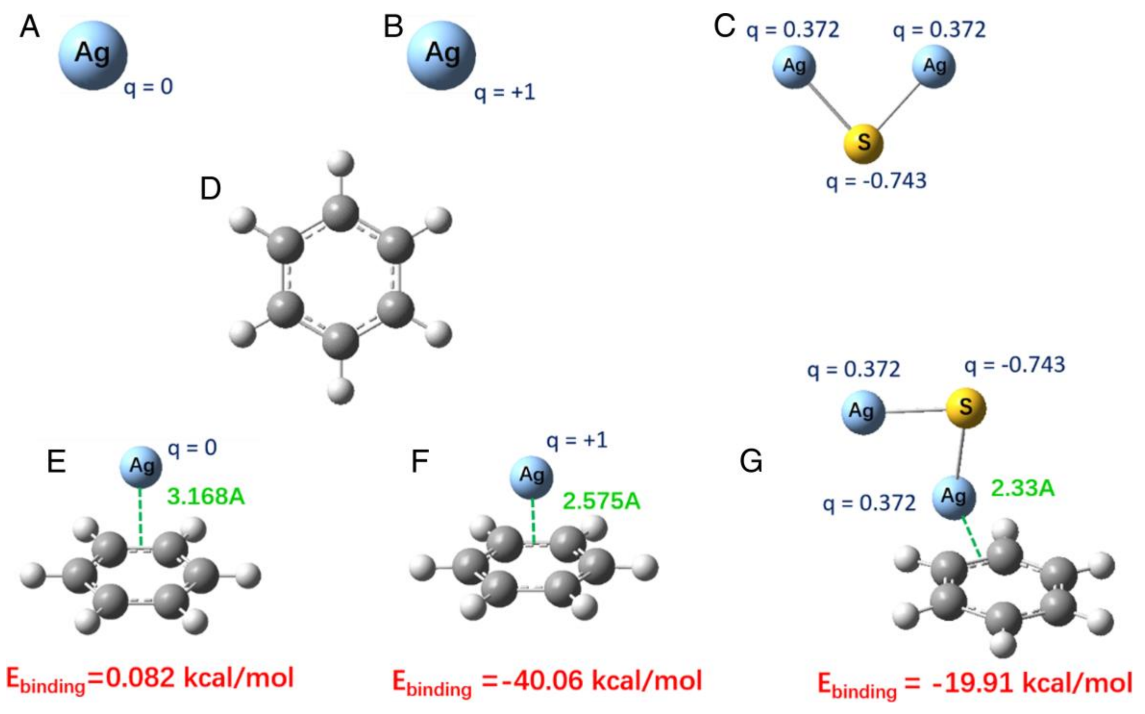

Figure S15. Structures of (A) Ag atom, (B) Ag (I) ion, (C) Ag_2S cluster, (D) phenyl and the binging energy of (E) Ag atom (F) Ag (I) ion and (G) Ag_2S cluster with the aromatic ring, related to Figure 3 and 4.

Transparent Methods

Materials: In this study, the positive nematic LC, SLC1717 ($\Delta n = 0.201$, $T_{NI} = 365$ K, $T_m = 233$ K) was purchased from Shijiazhuang Chengzhi Yonghua Display Material Co., Ltd and the polyvinyl alcohol (PVA) was from Alfa Aesar. The fluorescent dye, TPE-PPE was synthesized by a McMurry coupling and Sonogashira coupling, using the method of ref 13. The molecule structure is shown in Figure S6. All the chemicals used in the experiment were of analytical grade and without further purification.

Synthesis of amorphous Ag_2S hexagonal prisms: In a typical synthesis of the Ag complexes microrod, 4×10^{-5} mol sliver nitrate ($AgNO_3$) was first introduced into 30 mL dimethyl formamide (DMF) under constant stirring to obtain a homogeneous yellow solution at room temperature. Then the mixture was rapidly heated to 110 °C. Afterwards, 10 mL dimethyl sulfoxide (DMSO) was added dropwise and held until lawn green precipitate emerged. After being cooled down to room temperature, the product was centrifuged and washed with ethanol for several times. Then the yellow product was redisposed in ethanol and treated under visible light irradiation for 10 min to decompose to amorphous Ag_2S hexagonal prisms.

Preparation of LC/TPE-PPE composites: In this experiment, the LC/TPE-PPE composites were prepared by integrating TPE-PPE dyes with the concentrations of 0.01 wt% to 0.20 wt% intensively dissolved in dichloromethane into the systems of the nematic LCs. Then the generating solutions were sonicated for about 5 min to achieve excellent solubility. After full evaporation of the dichloromethane solvent from the above solutions, the uniform LC/TPE-PPE compositions were obtained accordingly.

Preparation of LC/TPE-PPE/Ag_2S composites: By injecting the as-prepared Ag_2S micro-rods dispersed in ethanol solvent into above LC/TPE-PPE composites, the composites with different Ag_2S mass ratios of 0.01wt% to 0.20 wt% were obtained. In order to yield an admirable dispersion in the isotropy of SLC1717, the composites were sonicated for about 5 min at 95 °C and were cooled down to the anisotropic phase while vivaciously stirring. The process alleviated aggregation resulted from the nucleation of nematic domains and is a fantastically crucial factor of preparing stable dispersions of micromaterials in LC systems. Then the compositions were placed for 1 day to evaporate off residual ethanol completely, and finally, the LC/TPE-PPE compositions contained only one type of microparticles and no other additives.

Fabrication of LC cells: The inner surfaces of transparent indium-tin oxide (ITO) glass plates were coated with a 3.0 wt% polyvinyl alcohol (PVA) layer by spin-casting. Then the ensuing substrates were dried at 80 °C for 30 min and rubbed with a textile cloth to impose boundary conditions for LC director N. Two prepared substrates were fabricated in anti-parallel directions and glued together

containing 10 μm PET (polyethylene terephthalate) films. Eventually, the studied compositions were respectively infiltrated into LC cells by capillary action.

Characterization: The powder X-ray diffraction (XRD) of the Ag_2S was characterized by the Rigaku Rotaflex Dmax2200 diffractometer with Cu Kα radiation (λ = 1.54056 Å). The morphologies of Ag_2S MRs were observed using Hitachi S-4800 scanning electron microscopy (SEM) with an accelerating voltage of 10 kV. Transmission electron microscopy (TEM) and High-resolution transmission electron microscopy (HRTEM) images were recorded by JEOL JEM-2100F, with an accelerating voltage of 200 kV. Optical textures of composites were observed by a polarizing optical microscope (POM, Olympus BX51, Japan). The absorption spectra of Ag_2S were obtained with a Shimadzu UV-VIS spectrophotometer (UV-2600). Photoluminescence (PL) spectra were characterized using a Shimadzu RF-5301 Fluorescence Spectrometer. Quantum yield were measured by Edinburgh FLS980 Fluorescence Spectrometer with 450 W xenon lamp and integrating ball accessories.
